# Investigating academic resilience in learning English: an ecological context of undergraduate students

**DOI:** 10.3389/fpsyg.2024.1467544

**Published:** 2025-01-29

**Authors:** Faiza Liaqat, Muhammad Islam, Muhammad Umer Azim, Ahmad Sohail Lodhi

**Affiliations:** ^1^Department of English Language and Literature, The University of Lahore, Lahore, Pakistan; ^2^Department of ELT and IER, University of the Punjab, Lahore, Pakistan; ^3^Department of English, Government Graduate College, Township, Lahore, Pakistan; ^4^Department of Business Education, IER University of the Punjab, Lahore, Pakistan

**Keywords:** academic resilience, English language learning, ecological system theory, ethnic differences, inclusive education

## Abstract

Despite a general understanding of the role resilience may play in students’ wellbeing and learning growth, there has been little or no focus on examining the resilient attitude of undergraduate L2 learners from Lahore, Pakistan. Students from various linguistic and cultural backgrounds are drawn to Lahore because it is a hub for higher education in Pakistan with almost 28 public and private sector universities. This quantitative study collected data from 498 undergraduate students from various academic disciplines and ethnic backgrounds studying in the universities of Lahore. The study utilized a survey questionnaire on Student AR in English Learning Scale, adapted to align with Bronfenbrenner’s Ecological System Theory. The study conducted statistical analyses, including Regression Analysis, one-way ANOVA, descriptive statistics, and frequency counts, to gain results. The findings underscored an important role of ecological factors, such as self-esteem, teacher support, peer support, and problem solving, which contributed significantly to the AR of participants. In addition, the findings also suggest that Baloch students exhibited lower scores across multiple dimensions of AR and social support as compared to all other ethnic groups, including Pashtun, Punjabis, Sindhi, and Saraiki. This research contributes to the understanding of resilience dynamics within a multicultural English language learning context and highlights the need to provide cultural sensitivity training to teachers and students in order to foster inclusive language learning environments.

## 1 Introduction

The study of Academic Resilience (AR) sheds light on how students use their strengths, such as self-control and empathy, as well as contextual assistance and support from both inside and outside their families, to overcome obstacles and disappointments (Rajik, 2022). The primary focus of AR literature is on finding the protective elements, both institutional and individual, that underpin the ability to overcome adversity and achieve academic achievement (Wills and Hofmeyr, 2019). Coping strategies and mechanism such as AR support students in overcoming obstacles, adjusting to classroom expectations, and succeeding in their academic endeavors. Language acquisition is a socialization process, “with learning and learners situated in complex environments” (Nolan and Owen, 2024). L2 learners’ interactions with their surroundings influence their academic performance from an ecological standpoint explicitly by the individual characteristics such as empathy and self-efficacy, they sustain (Griffiths and Soruç, 2021). This study focuses on AR as a coping strategy employed by undergraduate students from diverse linguistic and ethnic backgrounds against the challenges of learning English as L2 in the educational system of Lahore, Pakistan.

This study draws on Bronfenbrenner’s ecological system theory (EST) (Bronfenbrenner, 1979), which posits that human development is influenced by a complex interplay of different environmental systems. Due to the recognition that the surroundings in which second language learning takes place influence it, “context” has become more prominent and accepted in the field of L2 education (Chong et al., 2023; Li et al., 2020). EST employs a metaphorical use of the terms “ecology” and “ecosystem” to conceptualize contexts (Chong et al., 2023). EST framework is for understanding the four interacting layers of context that influence human development, including the immediate settings of students (microsystem) and interactions between factors within the microsystem influence broader contexts (mesosystem, exosystem, macrosystem) (Bronfenbrenner, 1979). EST can be used to examine single factors, groups of factors or whole systems as well as the relationships therein (Nolan and Owen, 2024). This study not only focuses on students’ AR but also rationalized the factors that interact to develop and sustain it, by incorporating EST. This study examines how undergraduate students employ coping mechanisms and resilience tactics during challenging circumstances within a multicultural microsystem, even if it is not specifically focused on language learning methods.

Lahore, a hub for higher education with nearly 28 public and private universities, attracts students from various linguistic and ethnic backgrounds. These students frequently need help to retain their heritage languages and cultures (Abrar-ul-Hassan et al., 2016) while learning another language. Students’ ability to navigate between home language and English dominated academic spaces is affected by the interaction between university and home environment. A disconnect between this mesosystem can increase feelings of isolation, especially among students from linguistic minorities. According to Abbas and Bidin (2022), Pakistani government policies have prioritized English and Urdu over regional languages, including Punjabi, Sindhi, Balochi, Pashto, and Saraiki. In certain regions, heritage languages are the fundamental components of ethnic identity; in others, they are frequently disregarded, particularly in Punjab (Abbas and Bidin, 2022). Due to an emphasis on competence in English, language standards have rapidly increased, endangering heritage languages and the ethnic identities connected to them, that may develop a suffocated exosystem that directly influences students’ wellbeing.

Undergraduate students in Pakistani universities encounter numerous challenges when learning English language. These difficulties range from deeper psychological issues such as fear and self-doubt to fundamental language skills like grammar, vocabulary, and pronunciation, significantly impeding their progress (Tayyab et al., 2023). For instance, English language learners at the University of Lahore, Gujrat campus often struggle with interlingual prepositional errors, especially when employing the “preposition of place” (Shafique and Mahmood, 2022). Khan et al. (2023) highlight significant obstacles for undergraduate students of English majors, such as the lack of a supportive environment for speaking practice and the low priority given to speaking and listening in the classroom, resulting in general deficiencies in English language proficiency. These challenges can be further understood within an ecological systems framework, where the immediate support of peers and teachers (microsystem) and broader societal expectations (exosystem and macrosystem) interact to shape personal traits like self-esteem, problem-solving skills, and empathy, which students rely on to adapt AR as a coping strategy. As educators, researchers, and policymakers, it is crucial to understand and address the challenges undergraduate students are facing in regard of their L2.

L2 learners are integral components of an educational system that includes practices and policies at the institutional, national, and international levels, in addition to a variety of interactions with necessary parties, including parents, friends, teachers, and other community members. All these parties contribute to building an ecological system and context around a learner (Wills and Hofmeyr, 2019). Moreover, research on student AR often emphasizes its strong connections with teachers’ belief in their effectiveness (Cassidy, 2015). Students demonstrate endurance and perseverance, often overcoming obstacles with the help of contextual support from their families and communities and through personal traits such as self-control and empathy (Lereya et al., 2016).

This study assumes that the combination of several internal and external factors within the exosystem of a multicultural context may impact students’ AR. Institutional policies and education support systems, along with cultural ethos that compels students with linguistic minorities develop AR have not been addressed directly in this research, the researchers contend that using the settings for university-level students in Lahore from diverse provinces of Pakistan, for example, a few external variables might affect how students cope with their L2 learning challenges and how they perceive it in a multicultural and multilingual demographics. In the context of English language education in Pakistan, focusing on potential ethnic differences, this study attempts to develop more sensitive inclusive and equitable language teaching environment and support structures for students with minorities languages, who are working hard to fulfill rigorous English language requirements.

The following research questions will guide this study:

**RQ1.** What factors contribute to academic resilience in learning English among undergraduate students in Lahore, Pakistan?

**RQ2.** What are the potential ethnic differences based on academic resilience in learning English of undergraduate students from Lahore, Pakistan?

## 2 Research design and methodology

This study has utilized a quantitative research approach. The quantitative data was collected through a survey questionnaire given to undergraduate students. The data was analyzed using SPSS (Statistical Package for the Social Sciences), version 20. For a detailed statistical analysis aligned with the research questions, one-way ANOVA, Multiple Regression analysis, McDonald’s omega, descriptive statistics, percentages, and frequency counts were applied. The data also gave a succinct picture of the participants’ demographic traits as well and perceived AR in English Language Learning settings.

### 2.1 Research participants

The participants are drawn from the top 10 universities in Lahore, as reported by Scimago Institutions Rankings, published 06 March, 2024. Approximately 123,458 undergraduate students are enrolled at these universities overall, according to information gleaned from their websites and validated by reaching out to the appropriate departments. The data included 498 undergraduate students, with 237 male participants making up 47.7% of the sample and 261 female participants constituting 52.2% of the sample. This distribution indicates a relatively balanced representation of both male and female participants.

The distribution of ethnic groups in this study, based on the six major ethnic groups in Pakistan, as outlined in the Central Intelligence Agency 2024, is significant. We employed convenience sampling and circulated the survey by approaching students from underrepresented ethnic backgrounds through their WhatsApp community groups. Without making any claims to generalizability, it may be reported that researchers made efforts, recognizing the geographical dominance of Punjabi students, to include minorities such as Pashtuns, Baloch, Sindhi and Saraiki, to ensure a more balanced representation of various ethnic groups. Despite these efforts, 64.3% of the sample identified themselves as Punjabis, Pashtuns (12.2%), and Saraikis (9.2%). Sindhis (2.8%) and Other (4.8%) have relatively small representation.

The above-mentioned distribution highlights the predominant representation of Punjabi students in our sample, as Punjab is home to the most significant number of degree-granting institutes, followed by the Federal Capital, Sindh, Khyber Pakhtunkhwa (KPK), Balochistan, Azad Jammu and Kashmir (AJK), and Gilgit-Baltistan (GB), according to the Higher Education Commission’s (HEC) Annual Report 2021–2022. Admission process in the universities under observation, for students coming from other provinces is hegemonized by quota system. Students other than Punjab are required to be nominated by the regional boards in their respective provinces rather than direct applications, specifically in government universities. This quota system is meant to ensure geographical and ethnic diversity. However, this study, despite researchers’ efforts, could manage only a reasonable number of participants from diverse ethnic groups outside Punjab.

There is another observation that is worth mentioning in relation to the status of education and inclusive accessibility of educational resources in other provinces for all genders. The data revealed, no female participants from Baluchistan. The researchers approached to the representative of the Baloch community WhatsApp group, who informed, based on their knowledge, that no female students from Baluchistan are currently studying in Punjab in any discipline. This indicates the issue of access and liberty available to Baloch female students for studying outside their province.

### 2.2 Research instrument

The questionnaire used in this study is a comprehensive tool that comprises two sections. The first part collects participants’ demographic information, aiming to ensure a thorough understanding of the sample. The second section is about Student Academic Resilience in English Learning Scale (SARELS) developed by Duan et al. (2024), which is based on the theoretical framework of Lereya et al. (2016). This 10-factorial structure of the resilience scale includes family connection, school connection, community connection, home and school life participation, community life, self-esteem, empathy, problem-solving, and peer support. This model, which encompasses individual characteristics and external supports, is widely used in studies on AR (Liu and Han, 2022; Shen, 2022; Zhang B., 2022), providing a robust foundation for this research.

Duan et al. (2024) excluded community factors such as community connection and participation in community life to better fit the context of language learning in China. Duan et al. (2024) highlighted the importance of individual traits, family support, teacher support, and peer support as integral components of students’ AR. His eight-factor model includes self-esteem, empathy, problem-solving, goals and aspirations, peer and teacher support. He emphasized the crucial roles of parental and teacher support in students’ AR and overall development, noting their contextual appropriateness.

For this study, student academic resilience in learning English scale by Duan et al. (2024) has been further modified by excluding goals and aspirations, aligning each dimension with Bronfenbrenner’s Ecological Microsystem framework. Goals and aspirations typically relate to future-oriented plans, ambitions, and expectations influenced by broader societal and environmental factors rather than immediate, direct interactions (Cheng, 2023). Focusing on the microsystem and excluding the exosystem-related “goals and aspirations,” the study remains tightly aligned with Bronfenbrenner’s theory, contextual relevance, and practical research needs. This approach ensures a clear, actionable, and contextually appropriate understanding of the factors contributing to AR in English learning among undergraduate students. Table 1 shows the dimensions of the Student Academic Resilience in English Learning Scale (SARELS), aligning each dimension with Bronfenbrenner’s Ecological Microsystem framework (Bronfenbrenner, 1979).

**Table 1:** SARELS within ecological microsystem.

Dimensions	Items distribution	Sources	Ecological microsystem (Bronfenbrenner, 1979)
Peer support (PS)	PS01, PS02, PS03, PS04	Lereya et al. (2016) and Liang (2017)	Support from peers, which occurs in the immediate environment of the individual, such as the classroom or social groups.
Teacher support (TS)	TS01, TS02, TS03, TS04, TS05, TS06	Liu and Li (2023)	Support from teachers, representing the direct interactions and relationships between students and their teachers in university.
Self-esteem (PC)	PC01, PC02, PC07	Lereya et al. (2016)	Individual’s perception of their own abilities, which are influenced by immediate interactions with peers, family, and teachers.
Empathy (PC)	PC03, PC04, PC08, PC09	Lereya et al. (2016) and Kim and Kim (2016)	Individual’s ability to understand and share the feelings of others, shaped by close interactions within the microsystem.
Problem-solving (PC)	PC05, PC10, PC11, PC12	Lereya et al. (2016)	Individual’s ability to resolve challenges, often influenced by immediate social interactions and support systems.
Family connection (FS)	FS01, FS02, FS03	Lereya et al. (2016)	Strength and quality of the individual’s relationship with family members, directly within the home environment.
Behavioral support (FS)	FS04, FS05, FS06, FS07, FS08	Liu (2013) and Liu (2023)	Behavioral support provided by family members, reflecting the immediate and direct influence of family on the individual.

### 2.3 The significance of referencing ecological systems theory in statistical analysis

EST is traditionally applied to qualitative and observational studies to understand how these environments interact and influence development. Statistical findings of the study through an ecological lens may develop an understanding of how different factors (such as self-esteem, empathy, and peer support) interact within the immediate settings of students (microsystem) and how these interactions influence broader contexts (mesosystem, exosystem, macrosystem). The ecological environment of Lahore, Pakistan, has already been hypothesized in 3.2. Statistical analyses reveal relationships between variables (such as AR and ethnic backgrounds). The study focuses on statistical analysis, and referencing EST enhances the findings’ ecological validity. It helps contextualize statistical relationships within real-world environments and interactions, providing a richer understanding of how AR operates within the multilingual and multicultural contexts.

### 2.4 Data collection

The data was collected through convenient and cluster sampling. Clusters of ethnic groups under study were approached through their WhatsApp community groups. Questionnaires were reviewed for completeness, consistency, and respondent eligibility. The quality of the responses was also assessed by excluding responses that appeared random or inappropriate.

## 3 Data analysis

First, we used Cronbach’s alpha to assess the internal consistency of the items within each factor category to ensure dependability. The Cronbach’s alpha values for every factor category exceeded 0.80, indicating good internal consistency and reliability. McDonalds’s omega (W) was also calculated as accounts for the dimensionality of the measured constructs for a more accurate estimate of internal consistency. The omega values for each subscale were consistent with Cronbach’s alpha. This suggests that the items within each category accurately assess the corresponding constructs.

### 3.1 Central tendencies and variability

Descriptive statistics revealed the central tendencies (mean) and variability (standard deviation) of seven factors, including peer support, teacher support, self-esteem, empathy, problem-solving, family connection, and family behavioral support, all of which contribute to AR.

The findings align with EST, which suggest that the individual development is shaped by multiple layers of environmental systems (Bronfenbrenner, 1979). The microsystem involves direct, intimate interactions that an individual has with their environment. In this study, family behavioral support, peer support, and teacher support, contribute directly to students’ AR, developing a microsystem. In the EFL context, the teaching and learning environments and educators’ relationships play a key role (Wang et al., 2024). Students reported moderate levels of peer support (Mean = 3.4233), yet relatively higher levels of teacher support (Mean = 3.8025), highlight the significant role that teachers are playing in fostering AR. This study reports the moderate level of family connection needs to be enhanced to bridge some gaps in the support system within the microsystem to foster resilience as a coping strategy. Moreover, moderate level of family connection and peer support can affect learners’ individual traits and subsequently their performance.

However, personal characteristics such as self-esteem (Mean = 3.8574), problem solving skills (Mean = 3.8851), and empathy (Mean = 3.9789) are indicating strong individual traits of learner and their capability to interact within environmental systems. The strong teacher support, family connections and individual characteristics of the students provide the background context of cultural ethos and policy frameworks of overall macrosystem of undergraduate students in Lahore. English as a sole medium of instruction in Higher education institutes and policy support for English education influenced how teachers, families and peers view language learning and how much they invest in it. These policies also affect how English language learning is perceived and supported.

### 3.2 Correlation matrix

The correlation matrix displayed the Pearson correlation coefficients between different dimensions of AR among undergraduate students. Peer support showed significant positive correlations with teacher support (*r* = 0.576, *p* < 0.01) suggests that these two key components of the microsystem reinforce one another. A positive and collaborative learning environment is crucial for resilience in language learning contexts. Similarly, the significant correlations between family connection and other dimensions such as self-esteem (*r* = 0.402, *p* < 0.01), problem-solving (*r* = 0.474, *p* < 0.01), and behavioral support (*r* = 0.647, *p* < 0.01) show that strong family bonds and behavioral support provide the emotional and practical foundation students need to thrive academically as a key support system in the microsystem.

The significant correlation between problem-solving and both peer support (*r* = 0.500, *p* < 0.01) and family connection (*r* = 0.474, *p* < 0.01) illustrates how both home and peer macrosystem contribute to the development of problem-solving skills in students. When families and peers actively engage with students, they foster the cognitive and emotional tools students need to tackle academic challenges. The positive relationships between dimensions such as self-esteem, empathy, and problem-solving (*r* = 0.663, *p* < 0.01) reflect how cultural attitudes toward personal responsibility and academic success may influence these traits. In societies where resilience, hard work, and academic success are culturally valued, students may feel a greater drive to develop these qualities. The macrosystem thus shapes the broader environment in which resilience is fostered. Self-esteem is positively and significantly correlated with Peer Support (*r* = 0.436, *p* < 0.01), Teacher Support (*r* = 0.543, *p* < 0.01), Empathy (*r* = 0.438, *p* < 0.01), Problem-solving (*r* = 0.663, *p* < 0.01), Family Connection (*r* = 0.402, *p* < 0.01), and Behavioral Support (*r* = 0.405, *p* < 0.01). This means that higher levels of self-esteem are associated with higher levels of peer support, teacher support, empathy, problem-solving abilities, family connection, and behavioral support among students. The findings show most of the items of AR are self-regulatory and adaptive, completely dependent on the external factors of their ecological system. It reinforces students’ ability to manage challenges, stay focused on academic goals, and develop problem-solving skills.

Empathy promotes positive relationships, cooperation, and emotional support within the microsystem (Wang et al., 2024). It contributes to a supportive mesosystem where students feel understood, valued, and motivated to persist through academic challenges (Rezai et al., 2024). Practical problem-solving skills enable students to overcome obstacles, adapt to academic demands, and maintain motivation and engagement in learning. They enhance students’ ability to navigate academic setbacks and achieve academic success. These findings emphasize the momentous role of educators and families in promoting students’ resilience. Development of AR in not dependent on one factor but all of them working together to develop interpersonal and intrapersonal skills of individuals. By focusing on fostering one dimension for example Peer Support, educators can have a positive effect on other dimensions to improve overall AR in English learning contexts. AR is the product of multiple, interacting systems, with peer support playing a key role within the students’ immediate environment.

The exosystem in Pakistan encompasses societal structures and institutions that significantly influence students’ AR. In particular, the socioeconomic background of students plays a crucial role in shaping their English language learning experiences. Students from wealthier families have access to better educational resources, including private schools, tuition centers, and modern learning environments (Rahman, 2020). Their socio-economic advantage over students from government schools (with less academic resources and facilities) may possibly enhance these students’ self-esteem, problem-solving abilities, and peer support, as they benefit from more advanced educational settings and stronger social networks. In contrast, students from lower socioeconomic backgrounds often face greater challenges due to limited resources, overcrowded public schools, and fewer opportunities for extracurricular activities. These limitations can hinder their academic resilience, forcing them to rely more heavily on teacher support or peer networks to compensate for the lack of family resources.

### 3.3 Multiple regression analysis

To address RQ1, the study conducted Multiple Regression Analysis to understand the factors contributing to academic resilience in a specific and quantifiable way. MRA allowed the study to assess the relative contribution of each independent variable (factors like peer support, teacher support, self-esteem, empathy, problem-solving, family connection, and family behavioral support) to the dependent variable (academic resilience). This technique determined:

- which factors have the strongest impact on AR.
- The direction and strength of each factor’s influence (positive/negative)

The results show that some of the factors are likely to emerge as significant positive predictors of AR. Outcome of the analysis are as Self-esteem: *β* = 0.45, *p* < 0.01 (strong positive predictor), Teacher support: *β* = 0.30, *p* < 0.01 (moderate positive predictor), Problem-solving: *β* = 0.25, *p* < 0.01 (moderate positive predictor), Peer support: *β* = 0.15, *p* = 0.03 (weak positive predictor), Family connection: *β* = 0.10, *p* = 0.06 (non-significant), Empathy: *β* = 0.08, *p* = 0.10 (non-significant).

Self-esteem emerged as one of the strongest predictors of academic resilience, which implies that students with high self-esteem may be more confident to overcome the challenges of learning English in the context of this study. Teacher Support has also appeared to be a strong predictor of AR, which suggests that students who perceive greater support from their teachers are more likely to be resilient in their studies. Similarly, peer support seemed to an important contributor to the academic resilience of the participants of this study. These findings highlight that both teachers and peers may act as major actors/enablers in the ecology of L2 learning in this Pakistani context. They can create condition which would help learners sustain their academic resilience. In addition, problem-solving was also found to be a significant predictor of resilience. Family Connection and Empathy did not contribute significantly to the academic resilience of participants.

The regression model explains 65% of the variability in academic resilience among undergraduate students in Lahore, Pakistan. This indicates that the factors of peer support, teacher support, self-esteem, and problem-solving collectively contribute strongly to students’ ability to overcome challenges in English language learning. However, 35% of the variance remains unexplained, suggesting the potential influence of additional factors beyond the scope of this study. Though, it leaves room for future research to explore other unexplored dimensions.

### 3.4 One-way analysis of variance (ANOVA)

A one-way Analysis of Variance (ANOVA) was conducted to compare the mean scores of resilience factors on SARELS across six distinct ethnic groups. Before conducting the one-way ANOVA, normality tests were conducted for each scale to confirm the normal distribution of the data. The results showed that the data (*N* = 498) were generally distributed, with most skewness and kurtosis values falling within acceptable ranges (less than |3.0| and |10.0|, respectively) (Kline, 2023). The consistent significant *p*-values (*p* < 0.05) across all factors highlight the statistically significant differences in academic resilience dimensions between different ethnic groups, further emphasizing the influence of ethnicity on academic resilience.

The results from the ANOVA test show that there are significant differences among ethnic groups in various aspects of academic resilience.

#### 3.4.1 Self-esteem

Pashtun students reported the highest self-esteem (*M* = 4.3, SD = 0.7), while Baloch students had the lowest (*M* = 3.4, SD = 1.1). Tukey’s post-hoc test revealed significant differences between Pashtuns and Balochs. Balochs diverge with the lowest mean self-esteem score verifies the potential challenges they may encounter.

#### 3.4.2 Problem-solving skills

The mean problem-solving skills of Pashtuns (*M* = 4.3, SD = 0.7) are the highest, a promising indication of their potential to handle academic and personal challenges. Conversely, Baloch have the lowest mean problem-solving skills (*M* = 3.4, SD = 1.1), suggesting specific areas where targeted support could significantly enhance their problem-solving abilities. Tukey’s post hoc analysis showed clear differences between Pashtuns and Baloch with other ethnic groups showing intermediate scores.

#### 3.4.3 Peer support

There were no significant differences across ethnic groups in terms of peer support at the alpha level (*p* > 0.05). This suggested that the peer support mechanisms within the universities are similarly perceived by all ethnic groups at undergraduate level.

#### 3.4.4 Teacher support

Tukey’s test revealed that Pashtun students perceive the highest level of teacher support (*M* = 4.2, SD = 0.8) indicating positive relationships and strong support from their teachers. On the other hand, Balochi students perceive the lowest level of teacher support (*M* = 3.3, SD = 0.9), suggesting potential challenges in teacher-student interactions and support for Baloch students.

#### 3.4.5 Empathy

Pashtuns and Punjabis have the highest reported mean empathy (*M* = 4.3, SD = 0.6), showing strong understanding and compassion among Pashtun students. On the other hand, Baloch have the lowest mean empathy (*M* = 3.5, SD = 1.0) indicating potential differences in interpersonal sensitivity and understanding compared to other groups.

#### 3.4.6 Family connection and behavioral support

Family connection scores were consistent across ethnic groups, while behavioral support scores revealed significant differences. Pashtuns (*M* = 4.18, SD = 0.68) and Sindhis (*M* = 4.21, SD = 0.59) reported the highest levels of behavioral support, while Balochs and Punjabis had lower scores (*M* = 3.63 and *M* = 3.80, respectively).

## 4 Language, multicultural Pakistan and potential ethnic differences

The data analysis showed statistically significant ethnic differences in AR among undergraduate students in Lahore. An individual’s ethnic identity is just as significant as their gender, age, race, sexual orientation, or socioeconomic status. It is connected to their personal histories and experiences, which may influence the success or failure of cross-cultural communication (Zhou et al., 2022). In multicultural environments where English is a second language, statements expressing ethnic affiliation, such as “we do not speak English,” are typical in order to prevent marginalization with other ethnic groups (Zhou et al., 2022). This impression could also draw attention to how Lahore is regarded to be English-speaking. Statistical findings are aligned with EST to understand ecological layers in multi-cultural Lahore.

### 4.1 Microsystem: individual and immediate interactions

Microsystem focuses on individual-level traits and the immediate environmental factors that influence them. Resilient students have low levels of stress and unclear control and high levels of self-esteem, perseverance, and planning (Lereya et al., 2016). In the multicultural context of Lahore Pashtuns demonstrate the highest level of self-esteem which shows their positive self-reflection and perception about their worth and abilities. Balochs diverge with the lowest mean verifies the potential challenges they may encounter. These challenges may include cultural assimilation, social discrimination, and economic disparities. All of which affect their self-perception within the urban context of Lahore.

The mean problem-solving skills of Pashtuns are the highest, a promising indication of their potential to handle academic and personal challenges. Conversely, Balochs have the lowest mean problem-solving skills, suggesting specific areas where targeted support could significantly enhance their problem-solving abilities. The strong community ties and cultural resilience of Pashtuns in Lahore may be a beacon of hope, contributing to their robust problem-solving skills. Meanwhile, Baloch students may face language barriers, socio-economic disparities, or cultural adaptation challenges within Lahore, but with the right support, their problem-solving abilities can be improved.

The results of the mean scores of peer support among different ethnic groups from post-hoc Tukey’s HSD test following an ANOVA is creating a homogeneous subset. This means that there are no statistically emphasized differences in peer support scores between these groups at the alpha level of 0.05. Punjabis with 3.3307, Sindhis: 3.3750, Other: 3.3958, Saraikis: 3.4239, Balochs: 3.4453 and Pashtuns: 3.9208. Harmonic mean of the group sizes, 32.273, is also used due to unequal group sizes for a more accurate comparison. Kilday and Ryan (2022) examined two viewpoints that clarify the worth of peer support for students’ motivation. First, opportunities for social support are afforded by the caliber of peer connections and exchanges within an outside the classroom. Second, peers as socializing agents that is connected with students’ beliefs, values and objectives. Friendship, social status, and the norms and supportive culture that define the classroom peer group (Kilday and Ryan, 2022).

In our study result shows that peer support mechanism within university settings is equally effective across ethnic group. Literature suggest that students favor peers who share their ethnic background, even when they have chance to interact with friends from different ethnic groups (Lereya et al., 2016). This problem is not covered by items PS01, PS02, PS03, or PS04 on the Academic Resilience Scale. It is advised to conduct observational studies and interviews to get insight into students’ preferences for peer assistance that goes beyond ethnic disparities. Gaining further insight into the variations in Academic Resilience among different ethnic groups will be beneficial.

### 4.2 Mesosystem: interconnections between microsystems

Mesosystem focuses the interplay between broader educational reforms and individual academic skills. Pashtuns might benefit from cohesive support systems that integrate home and educational settings, facilitating their problem-solving capabilities. Baloch may face systemic challenges that are rooted in the national education policies and provisional acumens. Pakistani officials have recently revised the 2009 National Education Policy by implementing bilingual education in the country’s government elementary schools. The previous policy was based on monolingual approach. As a result, students are now required to master three languages in the early grades: English, Urdu, and their native tongue (Pashto, Balochi, Punjabi, and Persian). This shift can potentially enhance students’ English language skills and problem-solving abilities but it may also pose challenges.

Zhang W. (2022) proposed that language use by the teachers in English language classrooms should be examined from a wider ecological context, which includes both internal and external ecological contexts (the natural and social environments), and that it should not be restricted to traditional contextual studies. The interaction between students, teachers, home and community environments can develop a support system for the students belonging to minorities groups.

Factors such as community norms, media influences, and societal expectations in the mesosystem shape empathetic behaviors. A study conducted by Khanda et al. (2021) illustrated how English imperialism affected Baloch students. They concluded that, although being heavily used as a communication tool, English is neglecting the Balochi languages, leading to a decline in their usage and preservation. This decline is having a detrimental effect on the cultural and linguistic heritage of the Baloch people. English will either harm Balochi or lose its prominence and prestige if it maintains its imperialistic position over the country (Khanda et al., 2021).

The higher reported behavioral support score for Pashtuns and Punjabis could be attributed to the strong support they receive from their immediate environments, such as peers, teachers, and family members. It is also worth noting that cultural practices and norms heavily influence the availability and effectiveness of behavioral support mechanisms in universities students (Zhou et al., 2022). Pashtun students may benefit from communal support and collective responsibility ingrained in their cultural values, contributing to their higher reported family connection and behavioral support scores. Punjabis also benefit from cohesive support networks, equipping them with the necessary tools to navigate behavioral challenges. It is essential to acknowledge the influence of cultural norms on support systems and to strive for a deeper understanding and enhancement of support for all students, particularly those reporting lower levels of behavioral support, such as Punjabi students.

### 4.3 Exosystem: indirect environmental influences

Exosystem deals with the impact of national policies, sociolinguistic hierarchies, and institutional decisions that indirectly influence individual experiences in education (Nolan and Owen, 2024). Despite Pakistan’s intricate sociolinguistic landscape with almost 70 identified heritage languages (Rahman, 2020), the new country’s ruling elite implemented a hierarchical bilingual/multilingual language policy, designating English as the language of higher education and official correspondence, Urdu as the national language and a medium of instruction for public sector schools up to secondary education, and indigenous languages for domestic use (Syed, 2024). However, a study by Abrar-ul-Hassan et al. (2016) assessing the effect of English as the sole medium of instruction on academic achievement revealed unexpected outcomes. Contrary to expectations, the study revealed that this policy may not support all students in higher education and could potentially exacerbate socioeconomic class divides. However, this emphasis on English proficiency is accompanied by complex attitudes toward language, as highlighted by Ashraf (2023), who revealed societal ambivalences toward both English as a foreign language and Urdu as a national language.

Indirect environmental factors significantly shape students’ opportunities and challenges in accessing higher education. According to the Human Rights Commission of Pakistan (HRCP), as of 2021, there were over 12,000 cases of enforced disappearances in Baluchistan alone (HRCP, 2021). Baloch students’ self-esteem may also be significantly damaged by the unresolved issue of missing individuals in Baluchistan, which also negatively affects their access to educational opportunities. Individuals with such political and societal dynamics experience psychological stress, increased workloads, and overall pessimism. The Tukey B post-hoc test results also indicate that Balochs have the lowest mean self-esteem score, forming a distinct group.

On the other hand, Baloch students with lower reported teacher support may need help forming meaningful connections with teachers. Jamaldini et al. (2022) investigates most teachers and parents at the secondary level in Nushki District, Balochistan express dissatisfaction with teaching English. This discontent is mirrored in the students’ performance, as many fail to progress adequately in the subject, resulting in poor academic outcomes. The root of the issue lies in the teachers’ need for proficiency in English, which directly impacts their ability to teach the subject effectively to different ethnic groups. Consequently, the students’ performance in English could be better, perpetuating a cycle of underachievement.

Empathy on post-hoc Tukey’s HSD test provided means of Sindhis: 3.4643, Balochs: 3.4848, Saraikis: 3.8804 Punjabis: 3.9875 Other: 4.1458 and Pashtuns: 4.3279. The result highlighted two homogeneous subsets for the alpha level of 0.05. Sraaikis are the part of both subsets with not a significant different score from either the lower group (Sindhi, Balochs) or the higher group (Punjabis, Other, Pashtuns). Hence, Saraikis presents an interesting case indicating a median position. The most empathetic Pashtuns and Punjabis may have the potential to preserve hospitality, decency, and compassion. Balochs, on the other hand, could struggle with interpersonal sensitivity. It’s possible that various ethnic communities have distinct definitions of empathy. It is advised to contextualize our knowledge of empathy on an academic resilience scale.

### 4.4 Macrosystem: cultural and societal context

Macrosystem discusses global and societal-level trends, such as the commodification of English, the sociocultural hierarchies. English, which was formerly only spoken by those who were native speakers of the language, is now spoken across the world, with non-native speakers making up 75% of all English speakers (Tauchid et al., 2022). This global spread of English has led to an expectation for non-native speakers to attain native-like proficiency, blurring the definition of “native” and raising questions about the importance of native-speaker status (Boonsuk and Ambele, 2020). This expectation occasionally results in non-native English speakers being perceived as less competent or inferior (Baese-Berk et al., 2020). In Pakistan, English has evolved as a cornerstone of academic and professional success, with proficiency in the language often perceived as essential for accessing higher education and competing in the global labor market (Rahman, 2020; Fareed et al., 2021). According to Haidar and Fang (2019), English is no longer merely a tool for communication but rather a commodity that confers social status and reputation. People invest in English, and it pays them in marketable, prominent employment. As a result, they become commodities to neoliberal thinkers (Haidar and Fang, 2019).

Heritage languages are not just the emblem of a community, but also its identity. Heritage languages may also reinforce cultural identities of individuals and. Since language serves as a channel for culture, ethnic ecological culture should be the first to be protected in terms of local/heritage language. Pashtuns may benefit from the cultural values prioritizing respect for their own languages and the monolingual culture of universities in Lahore, Pakistan. Thereby they have more recognition and commutation feasibility with authority figures, fostering positive teacher-student relationships. Teacher support mechanism are also dependent on national educational policies where heritage and local languages are already cornered.

The relationships among empathy, social support, and AR in the mental health domain are intricate and multidimensional. According to Zhou and Zhang (2021), students’ academic self-efficacy beliefs are directly correlated with their perceptions of the support they receive from their significant others (social support) and their ability to bounce back from setbacks (resilience). Rahman (2020) draws attention to the fact that Sindhi and Balochi, while being among the six official languages spoken by 3.57% of the population, are often marginalized in the political and economic spheres, underscoring the need for their preservation. How educational institutions treat students’ heritage languages influences their placement in the classroom system, depending on national legislation.

The challenges undergraduate students face during their transition from secondary to higher education depend on a student’s capacity for change management (Gale and Parker, 2014). These challenges fall into three major categories: intellectual, socioemotional, and personal (Sanagavarapu et al., 2019; Tsang, 2023). This shift may also be difficult for students who are unable of making it, especially considering the distinctions between secondary school and university environments. Universities in Lahore strictly follow the English Medium Instruction (EMI) policy that may multiply the challenges among undergraduate students who are coming from remote areas and other provinces with minimal integration of English language influence at secondary level.

Disproportions across the ethnic groups is quite significant which provide a clear roadmap for designing targeting need-based teaching methodologies to strengthen more all-encompassing environment in educational settings. Teachers at the university level must contextualize their instruction, connect it to ethnic literature and situations, and explain their beliefs to management and policy makers who must be persuaded of the advantages of inclusive education and possess a plan for its advancement.

## 5 Conclusion

These results suggest that Pashtuns as compared to Baloch, Punjabi Sindhi, Saraiki, and other ethnic students, generally have higher academic resilience and social support levels. In fact, consistently high mean scores for Pashtuns were found in self-esteem, problem-solving abilities, peer support, teacher support, empathy, and family connection. The most excellent mean behavioral support was also reported by Pashtuns and the least by Punjabis. Conversely, Baloch students are regarded as a lower-scoring ethnic group in terms of academic resilience and social support than other ethnic groups. As shown above, these findings underscore the importance to delve into and cater for Baloch students in Lahore based on their own unique educational needs. Tailored programs such as those that increased Baloch students’ self-esteem, problem-solving ability, teacher support (which is largely missing in the education system), empathy and family connection would greatly help.

## 6 Implications

Considering Baloch students’ lowest scores, here are some key recommendations and implications for improving their educational experiences and outcomes at university level in Lahore, Pakistan. These suggestions would lead to better education standards among all ethnic groups.

- Educational institutions should provide cultural sensitivity training to teachers and staff to better understand and support students’ unique cultural backgrounds and needs.
- Create and implement unique support plans to help Baloch students feel better about themselves and get better at solving problems. These plans might have mentors, someone to talk to for advice, classroom management and syllabus designs that help with personal growth.
- Foster better teacher-student interactions by encouraging open communication and creating a supportive classroom environment where Baloch students feel valued and understood.
- Work together with Baloch communities to boost how much families get involved in learning activities and support networks. This may include workshops for parents, sessions for family advice, and programs to reach out to them.
- Establish peer support networks or student groups where Baloch students can connect with peers facing similar challenges and share experiences and resources.
- Improving self-esteem, problem-solving skills, and support networks among Baloch students can positively impact their academic performance and overall educational outcomes.
- Promoting empathy and belonging in one’s family connection can help Baloch undergraduate students feel socially and emotionally competent. For years, this can improve the overall university culture in Lahore, Pakistan, by thwarting any ethnic group into isolation or marginalization.
- Influence policy creation from the grassroots academic levels to provide adequate resources, including support mechanisms for Baloch students and other marginalized communities. It may lead to stronger community relations and a more Inclusive and supportive educational community in Lahore.

Research on AR and wellbeing among different ethnic groups in Lahore, Pakistan, particularly the Baloch, should be approached comprehensively. Future research can consider other socio-cultural factors influencing AR and wellbeing among undergraduate students. Qualitative studies and mixed-method approaches are recommended for further research for an in-depth analysis of the effects of intersectional identities and ethnicities on educational experiences and outcomes.

## Data Availability

The raw data supporting the conclusions of this article will be made available by the authors, without undue reservation.

## References

[ref1] Abbas F. Bidin S. J. (2022). A critical analysis of the language planning and policy (LPP) in Pakistan and its impact on indigenous languages of Pakistan. Eurasian J. Appl. Ling. 8, 85–96. doi: 10.32601/ejal.911521

[ref2] Abrar-ul-Hassan S. Shahbaz M. Asif M. (2016). English medium instruction in higher education in Pakistan: policies, perceptions, problems, and possibilities. J. English Lingua Franca 5, 27–51. doi: 10.1515/jelf-2016-0002, PMID: 39220592

[ref3] Ashraf H. (2023). The ambivalent role of Urdu and English in multilingual Pakistan: a Bourdieusian study. Lang. Policy 22, 25–48. doi: 10.1007/s10993-022-09623-6, PMID: 35340722 PMC8939399

[ref4] Baese-Berk M. M. McLaughlin D. J. McGowan K. B. (2020). Perception of nonnative speech. Lang. Linguist. Compass. 14, 1–20. doi: 10.1111/lnc3.12375, PMID: 39710967

[ref5] Boonsuk Y. Ambele E. A. (2020). Who ‘owns English’in our changing world? Exploring the perception of Thai university students in Thailand. Asian Englishes 22, 297–308. doi: 10.1080/13488678.2019.1669302

[ref6] Bronfenbrenner U. (1979). The ecology of human development. Cambridge, MA: Harvard University Press.

[ref7] Cassidy S. (2015). Resilience building in students: the role of academic self-efficacy. Front. Psychol. 6:1781. doi: 10.3389/fpsyg.2015.01781, PMID: 26640447 PMC4661232

[ref8] Cheng X. (2023). Looking through goal theories in language learning: a review on goal setting and achievement goal theory. Front. Psychol. 13:1035223. doi: 10.3389/fpsyg.2022.1035223, PMID: 36687863 PMC9849896

[ref9] Chong S. W. Isaacs T. McKinley J. (2023). Ecological systems theory and second language research. Lang. Teach. 56, 333–348. doi: 10.1017/S0261444822000283

[ref10] Duan S. Han X. Li X. Liu H. (2024). Unveiling student academic resilience in language learning: a structural equation modelling approach. BMC Psychol. 12:177. doi: 10.1186/s40359-024-01665-1, PMID: 38539210 PMC10976839

[ref11] Fareed M. Jamal U. B. Zai R. A. Y. (2021). Peer feedback on writing skills: perceptions of Pakistani ESL postgraduate students. Pak. J. Educ. Res. 4, 399–415. doi: 10.52337/pjer.v4i1.169

[ref12] Gale T. Parker S. (2014). Navigating change: a typology of student transition in higher education. Stud. High. Educ. 39, 734–753. doi: 10.1080/03075079.2012.721351

[ref13] Griffiths C. Soruç A. (2021). Individual differences in language learning and teaching: a complex/dynamic/socio-ecological/holistic view. Engl. Teach. Learn. 45, 339–353. doi: 10.1007/s42321-021-00085-3

[ref14] Haidar S. Fang F. (2019). Access to English in Pakistan: a source of prestige or a hindrance to success. Asia Pacific J. Educ. 39, 485–500. doi: 10.1080/02188791.2019.1671805

[ref15] Jamaldini M. A. Jabbar A. Aminullah A. (2022). A study on the performance of students in English language education at public secondary schools of district Nushki, Balochistan. J. Dev. Soc. Sci. 3, 157–165. doi: 10.47205/jdss.2022(3-1)13

[ref16] Khan A. B. Ramanair J. Rethinasamay S. (2023). Challenges faced by Pakistani undergraduates and views of teachers about the challenges faced by their undergraduates in learning English using the collaborative learning approach (CLA). 3L: language, linguistics. Lit. Southeast Asian J. English Lang. Stud. 29, 186–198. doi: 10.17576/3L-2023-2902-13

[ref17] Khanda G. Ali Z. Brohi F. M. (2021). The impact of English language imperialism over Balochi. Jahan-e-Tahqeeq 4, 448–457. Retrieved from https://www.researchgate.net/publication/360540724.

[ref18] Kilday J. E. Ryan A. M. (2022). The intersection of the peer ecology and teacher practices for student motivation in the classroom. Educ. Psychol. Rev. 34, 2095–2127. doi: 10.1007/s10648-022-09712-2

[ref19] Kim T. Y. Kim Y. K. (2016). The impact of resilience on L2 learners’ motivated behaviour and proficiency in L2 learning. Educ. Stud. 43, 1–15. doi: 10.1080/03055698.2016.1237866, PMID: 39712432

[ref20] Kline R. B. (2023). Principles and practice of structural equation modeling. 5th Edn. New York, NY: The Guilford Press.

[ref21] Lereya S. T. Humphrey N. Patalay P. Wolpert M. Böhnke J. R. Macdougall A.. (2016). The student resilience survey: psychometric validation and associations with mental health. Child Adolesc. Psychiatry Ment. Health 10:44. doi: 10.1186/s13034-016-0132-5, PMID: 27822304 PMC5093941

[ref22] Li C. Abrar-ul-Hassan S. Gao F. (2020). An ecological perspective on university students’ sustainable language learning during the transition from high school to university in China. Sustain. For. 12:7359. doi: 10.3390/su12187359

[ref23] Liang X. (2017). Effect of teacher support and peer support on learner autonomy of English majors. Xi’an, Shaanxi, China: Xi’an International Studies University. 1–23.

[ref24] Liu H. (2013). Parental investment in junior high school students’ English learning: questionnaire design and its confirmatory analysis. J. Zhejiang Int. Stud. Univ. 28, 28–36.

[ref25] Liu H. (2023). Demystifying the relationship between parental investment in learners’ English learning and learners’ L2 motivational self system in the Chinese context: a Bourdieusian capital perspective. Int. J. Educ. Dev. 104:102973. doi: 10.1016/j.ijedudev.2023.102973, PMID: 39713500

[ref26] Liu H. Han X. (2022). Exploring senior high school students’ English academic resilience in the Chinese context. Chinese J. Appl. Ling. 45, 49–68. doi: 10.1515/CJAL-2022-0105

[ref27] Liu H. Li X. (2023). Unravelling students’ perceived EFL teacher support. System 115:103048. doi: 10.1016/j.system.2023.103048

[ref28] Nolan H. A. Owen K. (2024). Medical student experiences of equality, diversity, and inclusion: content analysis of student feedback using Bronfenbrenner’s ecological systems theory. BMC Med. Educ. 24:5. doi: 10.1186/s12909-023-04986-8, PMID: 38172809 PMC10765790

[ref29] Rahman T. (2020). “English in Pakistan: past, present and future” in Functional variations in English. Multilingual education. eds. Giri R. A. Sharma A. D'Angelo J. (Cham: Springer), 127–148.

[ref30] Rajik J. A. (2022). Second language lexical acquisition: the case of extrovert and introvert children. Psychol. Educ. Multidis. J. 4, 414–423. doi: 10.5281/zenodo.708775

[ref31] Rezai A. Soyoof A. Reynolds B. L. (2024). Ecological factors affecting students' use of informal digital learning of English: EFL teachers’ perceptions. Teach. Teach. Educ. 145:104629. doi: 10.1016/j.tate.2024.104629

[ref32] Sanagavarapu P. Abraham J. Taylor E. (2019). Development and validation of a scale to measure first year students’ transitional challenges, wellbeing, help-seeking, and adjustments in an Australian university. High. Educ. 77, 695–715. doi: 10.1007/s10734-018-0298-2

[ref33] Shafique H. Mahmood R. (2022). “Prepositional errors among undergraduate ESL learners in Pakistan” in English language teaching in Pakistan. eds. Ali Raza N. Coombe C. (Springer), 203–215.

[ref34] Shen Y. (2022). Mitigating students’ anxiety: the role of resilience and mindfulness among Chinese EFL learners. Front. Psychol. 13:940443. doi: 10.3389/fpsyg.2022.940443, PMID: 35865688 PMC9294629

[ref35] Syed H. (2024). Unravelling the deficit ideologies in English language education in Pakistan: a decolonial perspective. TESJ 15. doi: 10.1002/tesj.828, PMID: 39714112

[ref36] Tauchid A. Saleh M. Hartono R. Mujiyanto J. (2022). English as an international language (EIL) views in Indonesia and Japan: a survey research. Heliyon 8:e10785. doi: 10.1016/j.heliyon.2022.e10785, PMID: 36217467 PMC9547192

[ref37] Tayyab J. Hassan K. H. U. Akmal F. (2023). An exploratory study of issues of EFL learners in Pakistani University at Graduate Level. Pak. J. Human. Soc. Sci. 11, 196–209. doi: 10.52131/pjhss.2023.1101.0342

[ref38] Tsang A. (2023). The value of a semi-formal peer mentorship program for first-year students’ studies, socialization and adaptation. Act. Learn. High. Educ. 24, 125–138. doi: 10.1177/1469787420945212

[ref39] Wang G. Soleimanzadeh S. Elahi Shirvan M. (2024). An ecological perspective on the flow of compassion among Iranian learners of English as a foreign language. Stud. Second Lang. Learn. Teach. 14, 207–234. doi: 10.14746/ssllt.32804

[ref40] Wills G. Hofmeyr H. (2019). Academic resilience in challenging contexts: evidence from township and rural primary schools in South Africa. Int. J. Educ. Res. 98, 192–205. doi: 10.1016/j.ijer.2019.08.001

[ref41] Zhang B. (2022). The relationship between Chinese EFL learners’ resilience and academic motivation. Front. Psychol. 13:871554. doi: 10.3389/fpsyg.2022.871554, PMID: 35602676 PMC9121172

[ref42] Zhang W. (2022). Language, culture, and ecology: an exploration of language ecology in pragmatics. Engl. Lang. Teach. 15:80. doi: 10.5539/elt.v15n6p80

[ref43] Zhou E. Kyeong Y. Cheung C. S. Michalska K. J. (2022). Shared cultural values influence mental health help-seeking behaviors in Asian and Latinx college students. J. Racial Ethn. Health Disparities 9, 1325–1334. doi: 10.1007/s40615-021-01073-w, PMID: 34160819 PMC9249685

[ref44] Zhou J. Zhang Q. (2021). A survey study on US college students’ learning experience in COVID-19. Educ. Sci. 11, 1–17. doi: 10.3390/educsci11050248, PMID: 39659294

